# Rapid Field Response to a Cluster of Illnesses and Deaths — Sinoe County, Liberia, April–May, 2017

**DOI:** 10.15585/mmwr.mm6642a4

**Published:** 2017-10-27

**Authors:** John Doedeh, Joseph Asamoah Frimpong, Kwuakuan D.M. Yealue, Himiede W. Wilson, Youhn Konway, Samson Q. Wiah, Vivian Doedeh, Umaru Bao, George Seneh, Lawrence Gorwor, Sylvester Toe, Emmanuel Ghartey, Lawrence Larway, Dedesco Gweh, Philemon Gonotee, Thomas Paasewe, George Tamatai, James Yarkeh, Samuel Smith, Annette Brima-Davis, George Dauda, Thomas Monger, Leleh W. Gornor-Pewu, Siafa Lombeh, Jeremias Naiene, Nathaniel Dovillie, Mark Korvayan, Geraldine George, Garrison Kerwillain, Ralph Jetoh, Suzanne Friesen, Carl Kinkade, Victoria Katawera, Maame Amo-Addae, Roseline N. George, Miatta Z. Gbanya, E. Kainne Dokubo

**Affiliations:** ^1^Sinoe County Health Team, Liberia;^2^Liberia Field Epidemiology Training Program; ^3^World Health Organization, Liberia; ^4^National Public Health Institute of Liberia; ^5^Grand Gedeh County Health Team, Liberia; ^6^Grand Kru County Health Team, Liberia; ^7^Ricercess County Health Team, Liberia; ^8^Maryland County Health Team, Liberia; ^9^Ministry of Health, Liberia; ^10^CDC, Liberia.

On April 25, 2017, the Sinoe County Health Team (CHT) notified the Liberia Ministry of Health (MoH) and the National Public Health Institute of Liberia of an unknown illness among 14 persons that resulted in eight deaths in Sinoe County. On April 26, the National Rapid Response Team and epidemiologists from CDC, the World Health Organization (WHO) and the African Field Epidemiology Network (AFENET) in Liberia were deployed to support the county-led response. Measures were immediately implemented to identify all cases, ascertain the cause of illness, and control the outbreak. Illness was associated with attendance at a funeral event, and laboratory testing confirmed *Neisseria meningitidis* in biologic specimens from cases. The 2014–2015 Ebola virus disease (Ebola) outbreak in West Africa devastated Liberia’s already fragile health system, and it took many months for the country to mount an effective response to control the outbreak. Substantial efforts have been made to strengthen Liberia’s health system to prevent, detect, and respond to health threats. The rapid and efficient field response to this outbreak of *N. meningitidis* resulted in implementation of appropriate steps to prevent a widespread outbreak and reflects improved public health and outbreak response capacity in Liberia.

## Investigation and Results

Sinoe, one of 15 counties in Liberia, is located in the southeastern part of the country and has an estimated population of 102,391 (*1*). The county has 33 clinics and one hospital (F.J. Grante Memorial Hospital), which serves as the referral health facility. In the early hours of April 25, 10 patients were admitted to F.J. Grante Hospital in critical condition with symptoms including headache, altered mental status, generalized weakness, abdominal pain, vomiting, and diarrhea, which started after they attended a funeral event in Sinoe County on April 21 and 22. Five of the patients rapidly deteriorated and died within a few hours of admission. Three other patients with the same symptoms died before arrival at the hospital. Clinicians reported that a female patient aged 11 years with similar symptoms was admitted to the isolation ward on April 23 following illness onset the previous day and died a few hours after admission.

Sinoe CHT immediately notified national health authorities of the illnesses and deaths, and launched a county-led response with support from technical partners, using an Incident Management System that had been established during the 2014–2015 Ebola outbreak response (*2*). The multisectorial response comprised epidemiology/surveillance and data management, case management, infection prevention and control, laboratory, social mobilization and health promotion, psychosocial, and dead body management. All activities were coordinated by the County Health Officer and County Superintendent with support from technical partners; daily analyses and situational reports were shared with stakeholders.

Epidemiologic investigations were aimed at identifying all cases and establishing linkages and potential exposures. The investigation was reviewed in accordance with CDC’s human subjects review procedures and was determined to be nonresearch, routine public health activity. Based on symptoms reported among ill persons, a case was defined as the onset of two or more symptoms including headache, vomiting, mental confusion, or weakness, on or after April 10, 2017, in any person who had visited or lived in Sinoe County. A questionnaire was developed to gather demographic information, symptoms and onset date, as well as data on foods consumed, exposure to ill persons, travel history, and other potential exposures. Liberia Field Epidemiology Training Program–trained surveillance officers conducted case investigations and active case finding, with supervision from CDC, WHO, AFENET, and national-level epidemiologists.

**FIGURE 1:**
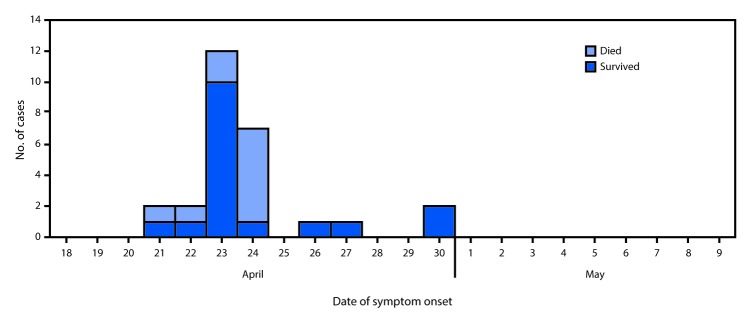
Number of cases of unexplained illnesses and deaths (N = 27) by date of symptom onset and outcome — Sinoe County, Liberia, April–May, 2017

**FIGURE 2:**
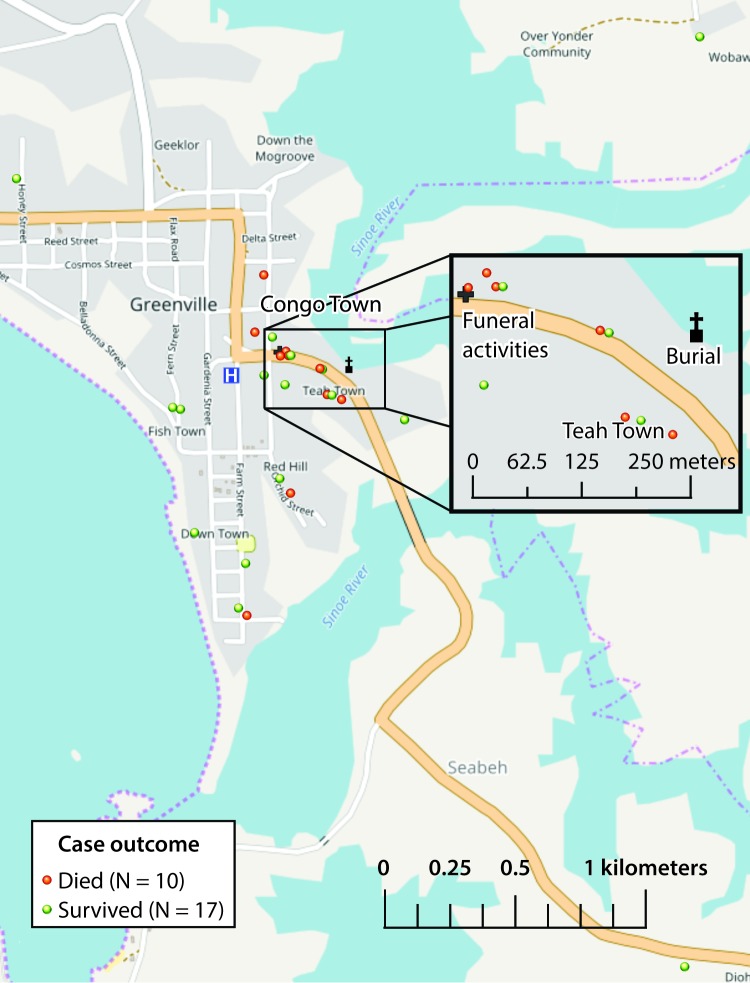
Unexplained cluster of illnesses and deaths: spot map of cases and location of funeral events — Sinoe County, Liberia, April–May, 2017

Twenty-seven cases were identified over the course of the investigation in Sinoe County; 16 (59%) occurred in females. The median patient age was 19 years (range = 10–54 years). The outbreak peaked on April 23 with 12 cases (Figure 1) and resulted in 10 deaths (case fatality ratio = 37%). Most cases were clustered around Teah Town community in Greenville city, Sinoe County (Figure 2), which recorded 11 cases with six deaths. A number of patients were family members or friends of one another, resided in the same household or neighboring houses, and attended the same school or place of worship. Information was also obtained from close contacts of patients and persons who attended the funeral but had not fallen ill.

The outbreak was hypothesized to be linked to attendance at funeral events on April 21–22 (overnight wake on April 21 and funeral service and burial in the early afternoon on April 22, followed by a repast in the late afternoon on April 22). A 1:2 unmatched case-control study with 25 case-patients and 50 controls was conducted to compare the odds of exposure among case-patients and controls (asymptomatic funeral attendees and community members). Statistically significant association with exposure was determined at a p<0.05. The inferential analysis showed that cases were 22 times more likely to have attended the funeral wake than were controls (odds ratio [OR] = 22.15, 95% confidence interval [CI] = 2.78–176.61) (p<0.05). Further analyses showed an association with food served at the wake, with the strongest association observed with consumption of tea (OR = 11.23, 95% CI 3.61–34.96). There were no reports of travel outside Liberia and no significant associations observed with other exposures analyzed.

Blood, urine, and stool specimens were collected from patients, and oral swab and pericardial fluid specimens were collected from decedents. Twenty-three specimens tested negative for Lassa fever and Ebola virus by reverse transcription–polymerase chain reaction at the National Reference Laboratory in Liberia. Hematologic and chemistry analyses of biologic specimens were unrevealing, and water samples from Sinoe County tested negative for coliforms. Food samples from the funeral event were collected for further analysis and biologic specimens from patients were sent to international laboratories for additional diagnostic evaluation.

## Public Health Response

Active case search and heightened surveillance in the community and health facilities were initiated to identify additional cases and persons at risk. Patients and their family members were interviewed by surveillance officers, clinical information was obtained from medical records, and a database was created to manage the epidemiologic, clinical, and laboratory data. In addition to the 27 cases, 60 funeral attendees and 152 contacts of cases in Sinoe County were identified and monitored daily.

Patients evaluated at the hospital were admitted to the isolation unit or emergency ward under close observation. Because the etiology of illness was initially unknown, clinicians provided supportive treatment, based on symptoms and physical examination findings of each patient, with intravenous fluids, supplemental oxygen, empiric antimicrobial therapy with broad spectrum antibiotics (including Ceftriaxone, Ciprofloxacin, and Metronidazole), and antimalarial therapy. Aside from one additional death that occurred on April 26, all seventeen subsequent patients who were managed at the hospital survived. Infection prevention and control standards and protocols were emphasized in health facilities and promoted in the community and at points of entry. Use of personal protective equipment by health care workers was reinforced, assessments for personal protective equipment availability were conducted, and adequate infection prevention and control supplies were provided to health facilities.

The social mobilization and health promotion team engaged community and religious leaders to raise awareness, dispel rumors, and overcome community resistance. Social mobilizers conducted house-to-house awareness activities and provided information to the public through radio talk shows and street broadcasters, encouraging ill persons to seek care at health facilities. A town hall meeting was convened to solicit information from community members and for health officials to answer questions from the public. Social mobilizers also accompanied surveillance officers during active case search, facilitating entry and surveillance activities in the community. The psychosocial team provided Psychosocial First Aid for bereaved families and conducted daily visits to patients who had been discharged from the hospital and patients who refused to seek care at the health facility. The dead body management team provided safe and dignified burials for decedents. Specimens collected from patients were transported daily via an existing sample transport network for testing at the National Reference Laboratory. Aliquots of specimens were sent to CDC for additional diagnostic evaluation and tested negative for heavy metals and organophosphates.

In addition to the 27 cases with 10 deaths reported in Sinoe County, four epidemiologically-linked cases, including three deaths, were reported in Montserrado and Grand Bassa Counties. The last death occurred on May 3 in Grand Bassa County and the last case linked to the cluster was reported on May 7. On May 8, Liberia MOH declared that the illnesses and deaths were attributable to a probable outbreak of meningococcal disease, based on detection of *N. meningitidis* in specimens tested at CDC headquarters in Atlanta.

## Discussion

Unexplained health events have significant implications when illness results from an infectious etiology and immediate control measures are not implemented (*3*). The presentation of cases and preliminary epidemiologic data suggested a common source outbreak or toxic exposure, and a concerted effort was made to rule out possible infectious etiologies. Building on response structures established during the Ebola epidemic (*2*), the CHT rapidly established all technical components of the response with support from the national rapid response team and technical partners, and was able to respond immediately to the health threat at its source, a requirement for an effective outbreak response (*4*).

The 2014 Ebola epidemic devastated Liberia’s already fragile health system, which was ill-prepared to respond to the initial cases of Ebola and prevent spread of infection (*5*,*6*). With support from international partners, the widespread outbreak was brought under control, and Liberia was declared free of Ebola virus transmission. The West Africa Ebola epidemic demonstrated that global health security relies on resilient health systems in all countries that are capable of rapidly detecting and controlling public health threats at their source (*7*). Support from CDC and other partners has increased capacity in core areas of disease surveillance, laboratory systems, workforce development and emergency operations, strengthening Liberia’s public health system against future disease outbreaks.

A robust surveillance system with capacity for immediate disease detection and reporting can facilitate response efforts and limit the magnitude of a potential outbreak (*8*). In Liberia, event-based surveillance for diseases of high epidemic potential or high morbidity and mortality is implemented through the Integrated Disease Surveillance and Response system, which captures 14 priority diseases and conditions, including unexplained clusters of health events and deaths (*9*). The prompt detection and immediate notification by district and county-level surveillance officers trained through the CDC-supported Liberia Field Epidemiology Training Program enabled the rapid response. Enhanced in-country laboratory capacity facilitated rapid testing and rule-out of Ebola and Lassa fever. Effective case management likely increased survival among patients, even before the diagnosis was confirmed.

Although the cause of illnesses and deaths was initially unknown, response measures were implemented while additional investigations were underway to determine the etiology and source of the outbreak. In 2014, an initial cluster of illnesses and deaths caused by Ebola took more than 90 days from detection to coordination of the emergency response and led to a widespread epidemic. In contrast, response efforts for this cluster of illnesses and deaths were initiated within less than 24 hours of detection. After effective control of the outbreak, WHO issued a notice on July 6, 2017, assessing the risk for recurrence of the meningococcal disease outbreak as low (*10*). Compared with the insufficient early response to the Ebola outbreak in Liberia in 2014, the rapid and effective response to this outbreak demonstrates the marked improvements in public health capacities in Liberia. Because public health emergencies such as Ebola and meningococcal disease outbreaks can rapidly spread internationally, these improvements in response capacity in Liberia contribute to enhance global health security.

**Summary**

**What is already known about this topic?**

The 2014–2015 Ebola virus disease (Ebola) outbreak in West Africa devastated Liberia’s already fragile health system, and it took many months for the country to mount an effective response to the epidemic. Substantial efforts have been made to strengthen Liberia’s health system to prevent, detect, and respond to future health threats.

**What is added by this report?**

In April 2017, a cluster of 27 cases of unexplained illness, including 10 deaths, occurred in Sinoe County, Liberia. Response measures were immediately implemented to ascertain the cause of illness, control the outbreak and prevent new cases and deaths. Epidemiologic investigations revealed that the cases occurred in persons who attended a funeral event in Sinoe County, and laboratory testing confirmed *Neisseria meningitidis* as the cause of illness. The Liberia Ministry of Health declared that the illnesses and deaths were attributable to an outbreak of meningococcal disease.

**What are the implications for public health practice?**

The rapid response to the cluster of illnesses and deaths is a reflection of the increased public health and outbreak response capacity established in Liberia during and subsequent to the Ebola epidemic, which has enhanced global health security. The response also highlights the importance of enhanced surveillance systems, improved laboratory capacity, a trained workforce and emergency management capacity to prevent widespread disease outbreaks.
